# Supplementary material for: WordCommentsAnalyzer: A windows software tool for qualitative research
Source: F1000Res. 2018 Sep 4;7:536. Originally published 2018 May 3. [Version 2] doi: 10.12688/f1000research.14819.2 (PMC6137411; doi:10.12688/f1000research.14819.2)
Supplement: Supplementary file 3 [file f1000research-7-17614-s0002.tgz › b2a79496-4ada-47c4-9682-a99ce867836d.zip!/Eight Iranian Health Journals/Journal of Health System Research _18.docx]

© Journal of Health System Research . This work is licensed under CC BY-NC 4.0 (https://creativecommons.org/licenses/by-nc/4.0/).

Volume & Issue: ویژه نامه آموزش بهداشت؛ 1392

Title: تأثیر برنامه آموزشی ترکیبی بر ارتقاء مراقبت از پادر بیماران دیابتی نوع 2 مراجعه کننده به کلینیک دیابت شهرستان سمیرم: کاربرد مدل بزنف

http://hsr.mui.ac.ir/index.php/jhsr/article/view/1695

چکیده مقدمه: پای دیابتی به عنوان یکی از عوارض دیررس دیابت ،هزینه زیادی  را به سیستم سلامت کشورها تحمیل می نماید.استراتژی رایج امروز در خصوص پیشگیری از پای دیابتی، آموزش مراقبت از پا است. مطالعه حاضر با هدف بررسی ارزشیابی برنامه آموزشی ترکیبی مبتنی بر مدل بزنف بر مراقبت از پا در بیماران دیابتی نوع 2 مراجعه کننده به کلینیک دیابت شهرستان سمیرم طراحی و اجرا شد. روش ها:پژوهش حاضر یک مطالعه نیمه تجربی و آینده نگر بود که164بیمار واجدشرایط پس از انجام پیش آزمون با انجام تخصیص تصادفی در دو گروه مداخله و مقایسه  وارد مطالعه شدند. روش جمع آوری داده ها ، بر اساس پرسشنامه محقق ساخته مبتنی بر سازه های مدل بزنف بود. قبل از مداخله آموزشی، اطلاعات دموگرافیک و مرتبط با سلامت و بیماری و چک لیست عملکرد مراقبت از پااز بیماران اخذ گردید. سپس افراد گروه مداخله، در برنامه اصول مراقبت از پا با استفاده از استراتژی آموزش ترکیبی که بر اساس سازه های مدل طراحی شده بود شرکت نمودند. افراد گروه مقایسه آموزش رایج کلینیک را طبق روال قبل ادامه دادند و به صورت جداگانه بدون تماس با گروه مداخله به تکمیل پرسشنامه ها و چک لیست، اقدام نمودند. پس آزمون در دو مرحله، 3و 6 ماه  بعد از مداخله آموزشی انجام گردید.داده ها با استفاده  از نرم افزار آماری SPSSنسخه 5/11و آزمون های آماری توصیفی و استنباطی مورد تحلیل قرار گرفت.سطح معنی داری در ین مطالعه کمتر از 05/0 در نظر گرفته شد. یافته ها:متغیرهای دموگرافیک در دو گروه تفاوت معنی داری نداشتند (05/0P>) . در گروه مداخله متغیر های آگاهی، اعتقادات رفتاری، نگرش نسبت به عمل،قصد رفتار، عوامل قادر کننده و عملکرد بیماران تفاوت معنی داری داشت(001/0P<). در گروه مقایسه این متغیرها اختلاف آماری معنی داری نشان ندادند (05/0P>). متغیر هنجارهای انتزاعی در دو گروه اختلاف آماری معنی داری نداشت(05/0P>). در مرحله سه ماه و شش ماه بعد از آموزش میانگین نمرات آگاهی، اعتقادات رفتار، نگرش، قصد رفتار، و عملکرد بیماراندر دو گروه اختلاف معنی داری داشت(05/0P<)، اما میانگین نمرات عوامل قادر کننده و هنجارهای انتزاعی در دو گروه مداخله و مقایسه اختلاف معنی داری نداشت(05/0P>).  نتیجه گیری: نتایج مطالعه موید این مطلب است که آموزش مبتنی بر مدل بزنف در ارتقاء رفتار مراقبت از پا در بیماران دیابتی مؤثر می باشد.بنابر این اجراء برنامه های آموزشی مبتنی بر این مدل ، پایش و پیگیری این برنامه ها در کلینیک های مراقبت از بیماران دیابتی پیشنهاد می گردد.

Title: رابطه ی مهارت های ارتباطی پزشکان و میزان رضایت بیماران سرپایی در درمانگاه های بیمارستان الزهرا سال 1390

http://hsr.mui.ac.ir/index.php/jhsr/article/view/1651

مقدمه : کیفیت مهارت های ارتباطی ارائه دهندگان خدمات بهداشتی درمانی تاثیر قابل توجهی بر نتایج درمانی بیماران دارد . تحقیق حاضر  به بررسی رابطه بکارگیری مهارت های ارتباطی در میزان رضایت مندی بیماران در درمانگاههای یکی از بیمارستانهای آموزشی استان اصفهان می پردازد. روش ها : این پژوهش یک مطالعه ی توصیفی – تحلیلی است و ابزار آن چک لیست استاندارد رضایت سنجی بیمار و چک لیست محقق ساخته مهارت برقراری ارتباط موثر بود. چک لیست توسط محقق در درمانگاه توسط بیماران یا همراهان آنها تکمیل گردید و به صورت تصادفی منظم نمونه گیری شد. چک لیست محقق ساخته مهارت برقراری ارتباط موثر با روایی صوری،ساختار و محتوا و پایایی(87% = α ) مورد تایید کارشناسان خبره قرار گرفت وپس از پاسخگویی بیماران بعد از ویزیت پزشک جمع آوری و با نرم افزار spss16و محاسبه ضریب همبستگی 2α و پیرسون تجزیه و تحلیل شد . یافته ها : مطالعه نشان داد که بین بکارگیری مهارت های ارتباطی در پنج حیطه کلامی، زبان بدن، شنود موثر، برقراری ، رعایت حریم خصوصی بیمار و مشارکت بیمار بع جزارتباط چشمیبا رضایت بیمار از پزشک ارتباط معنا دار(P-value < 0.05 )وجود دارد. نتیجه گیری : بکارگیری مهارت های ارتباطی توسط پزشکان ارتباط مستقیم با رضایت بیماران دارد و باعث افزایش پذیرش و مقبولیت پزشک از طرف بیمار می شود.لذا پیشنهاد می شود دربرنامه های آموزش های مداوم جامعه پزشکی فرصتی برای ارتقاء مهارتهای ارتباطی در کنار مهارت های بالینی فراهم شود .

Title: تأثير آموزش گروهي زوجين براساس رویکرد ارتباط درماني برافزايش سازگاري زناشویی زنان شهر قم

http://hsr.mui.ac.ir/index.php/jhsr/article/view/1183

مقدمه: ازدواج، يک نهاد اجتماعي است که پايه و اساس ارتباط انساني را تشکيل مي‌دهد. آن چه که در ازدواج مهم است سازگاري زناشويي و رضايت از ازدواج مي‌باشد. سازگاري زناشويي وضعيتي است که در آن زن و شوهر در بيشتر مواقع، احساس ناشي از خوشبختي و رضايت از همديگر دارند. سازگاري در ازدواج از طريق علاقه متقابل، مراقبت از يکديگر، پذيرش، درک يکديگر و ارضاي نيازها ايجاد مي‌شود. هدف اين پژوهش تعيين ميزان اثربخشي آموزش گروهی با استفاده از رویکرد ارتباط درمانی بر سازگاری زناشویی زنان شهر قم بوده است. روش‌ها: اين پژوهش نيمه تجربي است و از روش پيش آزمون - پس آزمون با گروه کنترل استفاده شده است. جامعه آماري شامل کليه زنان خود مراجعه کننده به مرکز مشاوره خانواده شهر قم در سال 90-91 بودند. روش نمونه‌گيري در اين تحقيق از نوع نمونه‌گيري داوطلبانه است و60 نفر از زنان به صورت تصادفي در دو گروه كنترل و آزمايش قرار گرفتند. در این پژوهش به منظور جمع‌آوری اطلاعات از پرسش‌نامه ویژگی‌های دموگرافیک و مقیاس سازگاری زناشویی استفاده شد. براي تجزيه و تحليل داده‌ها از نرم افزار SPSS با استفاده از روش‌هاي آمار توصيفي و تحليلی (کوواريانس) استفاده شد. يافته‌ها: نتایج پژوهش در تحلیل کوواریانس نشان داد رویکرد ارتیاط درمانی بر ابعاد رضایت زناشویی(05/0 P<، 01/3=F) همبستگی دونفره (05/0P<، 26/13=F)، توافق دو نفری (05/0 P<، 62/2=F) و ابراز محبت (05/0 P<، 88/5=F) تاثیر معنی‌دار داشته است ولی در سازگاری زناشویی (05/0 P>، 04/2=F) تاثیرنداشته است. نتیجه گیری: با توجه به نقش و اهمیت ارتباطات در رابطه همسران، این رویکرد می تواند با برطرف کردن موانع باعث بهبود و ارتقاء سازگاری زناشویی شود.

Title: بررسی همبستگی عملکرد انگیزشی سرپرستاران با سلامت روان پرستاران

http://hsr.mui.ac.ir/index.php/jhsr/article/view/1579

مقدمه: مطالعات نشان می‌دهد که درصدهای متفاوتی از پرستاران به انواع اختلالات و مشکلات روان شناختی مبتلا می باشند. ارتقاء سلامت روان مستلزم شناخت عوامل مرتبط با آن به منظور ترویج عوامل مؤثر و رفع فاکتورهای سو است. هدف از مطالعه حاضر، بررسی همبستگی عملکرد انگیزشی سرپرستاران با سلامت روان پرستاران بیمارستان های آموزشی قم می‌باشد. روش‌ها: اين مطالعه توصيفي و همبستگي است. 257 نفر از پرستاران سه بیمارستان آموزشی قم به‌ روش نمونه‌گيری در دسترس در سال 1392 مورد بررسی قرار گرفتند. ابزار جمع‌آوری داده‌ها پرسش‌نامه محقق ساخته ارزشیابی نحوه عملکرد انگیزشی سرپرستاران و پرسشنامه استاندارد سلامت عمومی 28سؤالی(GHQ-28)  با روش نمره‌گذاری ليکرت و نمره برش 23 بود. داده‌ها با استفاده از آمار توصيفي (ميانگين و انحراف ‏معيار) و آمار استنباطي (ضريب همبستگي پيرسون) تحت نرم افزار ‏SPSS‏ ‏نسخه 16مورد تجزيه و تحليل قرار گرفتند. يافته‌ها: نتايج نشان داد 1/45٪ پرستاران داراي سلامت روان و 9/54٪ در معرض اختلالات روان بوده‌اند. ميانگين نمرات پرستاران در مقياس اختلال در عملکرد اجتماعی نسبت به ديگر موارد بالاتر بود. نحوه عملکرد انگیزشی سرپرستاران از دیدگاه پرستاران در 7/37% موارد خوب و در 5/19% موارد ضعیف ارزشیابی شد. ‎همچنين نتايج نشان داد بين اختلالات روان پرستاران و تمامی زیر مقیاس ها با نحوه عملکرد انگیزشی سرپرستاران رابطه معکوس، ولی غیر معني دار وجود داشت (‏‎05/‎‏<‏P‏). نتيجه‌گيری: نقش انگیزشی سرپرستاران در ارتقا سلامت روان پرستاران مؤثر است ولی کافی نیست. حمایت بیشتر مدیران بیمارستان‌ها و سيستم‌های بهداشت و درمان کشور جهت برنامه‌ریزی اصولی با هدف پیشگیری، معاینات دوره‌ای و درمان اختلالات روانی، لازم و ضروری به نظر می‌رسد.

Title: ارزیابی آگاهی، نگرش و رفتار آموزگاران در مورد رفتارهای محافظت کننده در مواجهه با پدیده گرد و غبار بر اساس الگوی اعتقاد بهداشتی

http://hsr.mui.ac.ir/index.php/jhsr/article/view/1522

مقدمه:. پدیده گرد و غبار به دلیل ایجاد آثار نامطلوب بر سلامت یکی از مهم ترین نگرانی‌های بهداشتی است؛ این پدیده در اکثر مواقع سال شهر اهواز را با مشکلات جدی مواجه کرده است. معمولاً در هر جامعه‌اي تعداد مدارس در مقايسه با مراكز بهداشتي بيشتر است كه به راحتي مي‌توان از آنها در جهت ايجاد، آموزش و ترويج بهداشت استفاده كرد بنابراین این مطالعه با هدف تعیین آگاهی، نگرش و رفتار آموزگاران در مورد رفتارهای محافظت کننده در مواجهه با پدیده گرد و غبار بر اساس الگوی اعتقاد بهداشتی انجام گرفت. روش‌ها: در این مطالعه توصیفی- تحلیلی تعداد 200 آموزگار زن مدارس ابتدایی دخترانه شهر اهواز مورد تحقیق قرار گرفتند. ابزار گردآوری اطلاعات پرسشنامه محقق ساخته چند قسمتی شامل اطلاعات فردی، آگاهی، ادراکات و رفتار بود که روایی و پایایی آن قبل از مطالعه بررسی شد. داده‌ها با استفاده از نرم‌افزار SPSS نسخه 18 و آزمون‌های کای اسکوئر، ضریب همبستگی پیرسون و ANOVA در سطح معنی‌داری 05/0> P تجزیه و تحلیل شدند. یافته‌ها: یافته‌های پژوهش نشان داد ميانگين سني واحدهای مورد پژوهش 97/6 ± 76/39 سال بود. میانگین نمره رفتار 45/4 ± 56/33 به دست آمد. میانگین حساسیت درک شده 66/2 ± 2/27، شدت درک شده 19/2 ± 89/28، منافع درک شده 9/2 ± 42/28، موانع درک شده 56/4 ± 18/16 و خودکارآمدی درک شده 34/2 ± 79/12 به دست آمد. نتیجه‌گیری: طراحی و اجرای برنامه‌های مختلف آموزشی و مداخله‌ای در مدارس در ارتقای آگاهی در زمینه رفتارهای محافظت کننده در مقابل ذرات گرد و غبار ضروری به نظر می‌رسد.

Title: بررسی سازه های الگوی اعتقاد بهداشتی در خصوص اضطراب بارداری در زنان باردار نخست‌زا

http://hsr.mui.ac.ir/index.php/jhsr/article/view/1315

مقدمه: بارداری برای زن و خانواده او یک تجربه منحصر به فرد است. تغییرات فیزیولوژیک ناشی از بارداری علاوه بر جسم، روح و روان زن باردار را هم تحت تأثیر قرار می‌دهند. بنابراین خلق مادر تغییر کرده و احساسات کلی اضطراب در این دوران شایعند. با توجه به عوارض نامطلوب و جبران‌ناپذیر اضطراب بر مادر، جنین و پیامد بارداری، این مطالعه با هدف بررسی اضطراب در زنان باردار نخست زا مبتنی بر سازه‌های الگوی اعتقاد بهداشتی انجام شد. روش‌ها: این مطالعه توصیفی تحلیلی از نوع مقطعی می‌باشد. جامعه پژوهش در این مطالعه شامل 88 زن باردار نخست‌زای مراجعه کننده به درمانگاه‌های بهداشتی شهرستان دورود بودند که به روش نمونه‌گیری تصادفی انتخاب شدند. اطلاعات بر اساس یک پرسشنامه محقق ساخته که روایی و پایایی آن مورد تأیید قرار گرفت جمع‌آوری شد. اطلاعات جمع‌آوری شده وارد نرم‌افزار SPSS نسخه 20شده و با آزمون‌های آماری؛ T مستقل، همبستگی اسپیرمن، همبستگی پیرسون و آنالیز واریانس یکطرفه در سطح معنی‌داری کمتر از 05/0 تجزیه و تحلیل شدند. یافته‌ها: نتایج این مطالعه نشان داد که میانگین نمره آگاهی در خصوص اضطراب در زنان باردار نخست زا 05/24±  1/31 بود. همچنین در زنان باردار مورد بررسی؛ میانگین نمره حساسیت درک شده 90/29± 5/35، شدت درک شده 36/29±  03/29، منافع درک شده 44/26 ± 49/42، موانع درک شده 38/31 ±24/24، خودکارآمدی 94/31 ±07/30، راهنمای عمل 09/33± 22/ 22 و عملکرد 21/40 ± 88/29، به دست آمد. بر اساس نتایج این پژوهش، ارتباط معنی‌داری بین آگاهی زنان باردار نخست زا و سن آنها مشاهده شد. همچنین بین میزان تحصیلات و وضعیت اشتغال این زنان با سازه راهنمای عمل ارتباط معنی‌داری وجود داشت. نتیجه‌گیری: نتایج این مطالعه میزان آگاهی و نمرات پایینی را در سازه‌های مدل و همچنین عملکرد زنان باردار در خصوص اضطراب نشان داد و با توجه به اینکه بررسی اضطراب در بارداری به سبب بروز عوارض مختلف و جبران ناپذیر آن با اهمیت است، پیشنهاد می‌شود مسؤولین مربوطه در طراحی و اجرای برنامه‌های آموزشی بر اساس الگوی اعتقاد بهداشتی جهت کاهش اضطراب بارداری تلاش نمایند.

Title: پیشگیری از گرایش فرزندان به مواد اعتیاد آور: بررسی نقش هنجارهای ذهنی، اعتقادات هنجاری و انگیزه های اطاعت پدران

http://hsr.mui.ac.ir/index.php/jhsr/article/view/1312

مقدمه: خانواده به عنوان محل پایه‌ریزی شخصیت فرزندان نقش بسیار مهمی در پیشگیری از آسیب‌های اجتماعی، من جمله اعتیاد به مواد مخدر دارد. مطالعه حاضر با هدف تعیین نقش هنجارهای ذهنی، اعتقادات هنجاری و انگیزه‌های اطاعت پدران در پیشگیری از گرایش فرزندان به مواد اعتیاد آور صورت گرفت. روش‌ها:این مطالعه توصیفی- مقطعی در بین 180 نفر از پدران شهر یزد انجام شد. ابزار جمع آوری اطلاعات، پرسشنامه در سه بخش؛ هفت سوال جمعیت‌شناختی و زمینه‌ای، چهار گویه هنجارهای ذهنی (78/0=α) چهار گویه عقاید هنجاری (81/0=α) و چهار گویه انگیزه برای اطاعت (88/0=α) بود که به صورت خود گزارش دهی تکمیل می‌شد. داده‌ها توسط نرم‌افزار آماری SPSS نسخه 18 و با استفاده از آزمون‌های تی تست مستقل، آنووا، آنالیز رگرسیون خطی و ضریب همبستگی پیرسون در سطح معنی‌داری 05/0 مورد تجزیه و تحلیل قرار گرفتند. یافته‌ها: محدوده سن شرکت‌کنندگان 30 الی 59 سال با میانگین 34/5±38/40 بود. 8/22٪ دارای سابقه اعتیاد در خانواده و دوستان خود را گزارش کردند. در بخش هنجارهای ذهنی و اعتقادات هنجاری، تایید همسر و در قسمت انگیزه، اطاعت از بهترین دوستان امتیاز بالاتر کسب شد. در سازه‌های هنجارهای ذهنی، اعتقادات هنجاری و انگیزه اطاعت به ترتیب 2/78، 6/68 و 5/70٪ از حداکثر نمره قابل اکتساب کسب شد. پدران با سطح تحصیلات لیسانس و کارمند از انگیزه اطاعت بالاتری برخوردار بودند. اعتقادات هنجاری پیشگویی کننده قوی‌تر نسبت به انگیزه اطاعت بود و 42٪ از واریانس هنجارهای ذهنی را پیشگویی نمود. نتیجه‌گیری: به نظر می‌رسد در تهیه و اجرای برنامه‌های آموزشی در خصوص ترغیب پدران به انجام رفتارهای پیشگیری‌کننده از گرایش فرزندان به مواد اعتیاد آور تاکید بر نقش همسران و دوستان به تواند نتایج سودمندی را به دنبال داشته باشد.

Title: بررسی مهارت نوجوانان 14-11 ساله شهر تهران در زمینه مصرف میوه و سبزی: یک مطالعه مقطعی

http://hsr.mui.ac.ir/index.php/jhsr/article/view/1311

مقدمه: مطالعات متعددی مصرف کم میوه و سبزی را در نوجوانان گزارش کرده‌اند که مهارت به عنوان یکی از تعیین کننده‌های این رفتار محسوب می‌شود. این مطالعه با هدف بررسی مهارت نوجوانان تهرانی در زمینه مصرف میوه و سبزی در سال 1391 انجام شده است. روش‌ها: پژوهش حاضر، یک مطالعه مقطعي (Cross-sectional) از نوع توصيفي - تحليلي مي‌باشد. در این تحقیق 500 نفر از نوجوانان 14-11 ساله شهر تهران مورد بررسی قرار گرفتند. روش نمونه‌گيري به صورت تصادفي چند مرحله‌اي بود. معیار انتخاب نمونه‌ها، داشتن 14-11 سال سن و رضايت آگاهانه و مشاركت داوطلبانه دانش‌آموز در مطالعه بود. ابزار جمع‌آوری داده‌ها پرسشنامه محقق ساخته مشتمل بر مشخصات دموگرافیک و سؤالات مهارت بود که روایی صوری و محتوایی آن بررسی شده و پایایی نیز به روش همسانی درونی (67/0(α=  سنجیده شد. براي تجزيه و تحليل داده‌ها در این تحقیق از نرم افزارSPSS 18  (آمار توصیفی، آزمون‌های t، کای دو و آناليز واريانس یک طرفه) استفاده گردید. یافته‌ها: ارتباط معنی‌داری بین جنس، شغل پدر و شغل مادر با مهارت نوجوانان در خصوص مصرف میوه و سبزی مشاهده نشد. همچنین ارتباط معنی‌داری بین سطح تحصیلات مادر و منطقه سکونت افراد با مهارت وجود داشت، اما در خصوص سن این ارتباط معنی‌دار نبود. نتیجه‌گیری: طراحی و اجرای مداخلات توانمندسازی و مهارت‌سازی نوجوانان در خصوص مصرف میوه و سبزی با مشارکت دانش‌آموزان، خانواده‌ها و معلمان توصیه می‌گردد.

Title: روانسنجی ابزار سنجش آگاهی تغذیه ای، ادراکات بیماری و پیگیری رژیم غذایی در بیماران پرفشاری خون

http://hsr.mui.ac.ir/index.php/jhsr/article/view/827

مقدمه: ابزارهای اندازه‌گیری طراحی شده در مطالعات برای اطمینان از دستیابی به اهداف در نظر گرفته شده، باید مورد ارزشیابی قرار گیرند. مطالعه حاضر با هدف تعیین روایی و پایایی پرسشنامه طراحی شده برای سنجش آگاهی تغذیه‌ای و ادراکات بیماری در بیماران پرفشاری خون انجام گرفته است. روش‌ها : در یک مطالعه توصیفی- مقطعی پرسشنامه طراحی شده با  بهره‌گیری از نظرات 11 عضو هیات علمی با سابقه و صاحب نظر و همچنین 671 پرسشنامه تکمیل شده توسط بیماران پرفشاری خون روستایی در سال 1392 مورد ارزیابی قرار گرفت. برای بررسی پایایی از شیوه همسانی درونی ( آلفای کرونباخ)، دونیمه سازی و کودرریچاردسون استفاده و روایی (اعتبار) پرسشنامه مزبور به شیوه روایی محتوا و سازه‌ای بررسی گردید. داده‌ها با نرم افزار SPSS نسخه 18 تجزیه و تحلیل شد. یافته‌ها : نتایج ضریب اسپیرمن براون، آلفاي كرونباخ، گاتمن و کودرریچاردسون در پرسشنامه در حد مطلوب و نشانگر رضايت بخش بودن پایایی اين پرسشنامه است. سؤالات پرسشنامه همبستگی بالایی با نمره کل دارند و بررسی همسانی درونی سؤالات نشان می‌دهد که سؤالات پرسشنامه از پایایی قابل قبولی برخوردار بودند. در تحلیل عاملی، تمامی سؤالات پرسشنامه دارای بار عاملی قابل قبول بودند و بر پایه تحلیل عاملی همه سؤالات پرسشنامه مهم بوده و دارای روایی سازه مناسب می‌باشند. همچنین این پرسشنامه با ضریب روایی بالای 9/0 مورد تایید اساتید بود. نتيجه‌گيري: پرسشنامه طراحی شده از خصوصیات روان سنجی مناسب برای اندازه‌گیری متغییرهای مدنظر در بیماران پرفشاری خون برخوردار است. پایایی و روایی پرسشنامه مطلوب و ساختار کلی سؤالات مورد تایید قرار گرفت.

Title: بررسی تاثیر برنامه آرامسازي پيشرونده عضلاني بر پارامترهای افسردگی، اضطراب و استرس دانشجویان دختر با سندرم پیش از قاعدگی

http://hsr.mui.ac.ir/index.php/jhsr/article/view/1209

مقدمه: سندرم پیش از قاعدگی یک اختلال روانی – عصبی – غددی است که سبب اختلال در ارتباطات، مختل شدن فعالیت‌های طبیعی، کم‌تحرکی، بی‌انگیزگی و کاهش دقت در انجام کارهای فرد می‌شود و در صورتی که شدت علایم و نشانه‌های آن زیاد باشد شیوه زندگی، آسایش و سلامت فرد را تحت تاثیر قرار می‌دهد. این سندرم به عنوان یک بیماری با ایجاد تغییراتی در مشخصات فردی و رفتاری دانشجویان دختر، تاثیر مهمی بر عملکرد تحصیلی آنان دارد. در همین راستا مطالعه حاضر با هدف بررسی تاثیر برنامه آرام‌سازي پيش‌رونده عضلاني بر پارامترهای افسردگی، اضطراب و استرس دانشجویان دختر با سندرم پیش از قاعدگی ساکن خوابگاه در دانشگاه اصفهان انجام پذیرفت. روش‌ها: مطالعه حاضر مداخله‌ای نیمه تجربی است، که درسال تحصیلی1390-1391 در خوابگاه‌های دانشگاه اصفهان انجام گرفت.آزمودني‌هاي پژوهش 64 دانشجوی ساکن خوابگاه بودند که در 2 گروه آزمون (32 نفر) و شاهد (32 نفر) به صورت تصادفی تخصیص یافتند. ابزار گردآوری داده‌ها شامل پرسشنامه‌های اطلاعات دموگرافيک، غربالگری سندرم پیش از قاعدگی (PSST) و DASS21 بود. برنامه آرام‌سازی پیش‌رونده عضلانی طی 6 جلسه در گروه آزمون اجرا شد و پس از 6 هفته در دو گروه پس آزمون به عمل آمد. اطلاعات حاصله با استفاده ازآزمون‌هاى آمارى تی مستقل، آزمون آنالیز واریانس با تکرار مشاهدات،کای اسکوئر و من ویتنی مورد تجزیه و تحلیل قرارگرفت. یافته‌ها: آزمون آماری کای اسکوئر و من ویتنی اختلاف معنی‌داری را از نظر متغیرهای دموگرافیکی بین دو گروه آزمون و شاهد نشان نداد. آزمون آنالیز واریانس با تکرار مشاهدات نشان داد که بین میانگین نمره سندرم پیش از قاعدگی قبل، بلافاصله و 2 ماه پس از مداخله در گروه شاهد اختلاف معنی‌داری وجود ندارد (05/0P>)، اما میانگین نمره سندرم پیش از قاعدگی در گروه آزمون را در زمان‌های مختلف دارای اختلاف معنی‌دار نشان داد (05/0>P). آزمون T مستقل نشان داد میانگین نمره اضطراب و استرس در زمینه سندرم پیش از قاعدگی دانشجویان در دو گروه، قبل از مداخله اختلاف معنی‌داری ندارند، اما بلافاصله و 2 ماه بعد از آموزش میانگین نمره اضطراب در گروه آزمون به طور معنی‌داری کمتر از گروه شاهد بود (05/0>P). در مورد نمره افسردگی در زمینه سندرم پیش از قاعدگی در دانشجویان، آزمون T مستقل قبل از آموزش در دو گروه اختلاف معنی‌داری را نشان نداد. همچنین 2 ماه بعد از آموزش علی‌رغم کاهش نمره افسردگی در گروه مورد این اختلاف معنی‌دار نبود (09/0 P=). نتیجه‌گیری: نتایج حاصله از پژوهش حاضر نشان داد اجرای برنامه‌های آرام‌سازی پیش‌رونده عضلانی در دانشجویان دختر دارای علایم سندرم قاعدگی، منجر به کاهش میزان اضطراب، استرس و افسردگی آنان شده است. با توجه به اینکه این دوره یکی از حساس‌ترین دوره‌های زندگی هر جنس مونث است و فرد توام با ناتوانی در انجام عملکرد اجتماعی و تحصیلی مواجه استواز سویی دیگر با توجه به ماهیت اینگونه برنامه‌ها که در عین ارزان و ایمن بودن، کارا و اثربخش نیز می‌باشند، اجرای آن‌را در جهت کنترل افسردگی، اضطراب و استرس دختران دانشجو توصیه می‌نماییم.

Title: پيشگويي کننده‌هاي خود مراقبتي و عوامل موثر بر آن در میان بیماران زن دیابتی زاهدان بر اساس مدل اعتقاد بهداشتی

http://hsr.mui.ac.ir/index.php/jhsr/article/view/1261

مقدمه: خود مراقبتی نقش مهمی در مدیریت موفق دیابت دارد، بنابراین برای کارکنان بهداشتی شناسایی عوامل موثر در خود مراقبتی بیماران دیابتی بسیار مهم است. مطالعه حاضر با هدف تعیین پيش‌گويي کننده‌هاي خود مراقبتي و عوامل موثر بر آن در میان بیماران زن دیابتی زاهدان بر اساس مدل اعتقاد بهداشتی انجام شده است.   روش‌ها: در این مطالعه مقطعی 180 بیمار زن دیابتی مراجعه کننده به مرکز دیابت زاهدان به روش چند مرحله‌ای انتخاب و وارد مطالعه شدند. ابزار گردآوری اطلاعات پرسشنامه‌ای شامل سازه‌های مدل اعتقاد بهداشتی و رفتارهای خود مراقبتی بیماران دیابتی بود که روایی و پایایی آن مورد سنجش قرار گرفت و به روش مصاحبه با بیماران تکمیل گردید. اطلاعات جمع‌آوری شده با استفاده از آزمون تی‌مستقل و رگرسیون چندگانه در سطح معنی‌داری کمتر از 05/0 مورد تجزیه و تحلیل قرار گرفتند. یافته‌ها: میانگین سنی شرکت‌کننده‌ها 24/47 با انحراف معیار 16/7 بود. خودکارآمدی، منافع و موانع درک شده پیشگویی کننده‌های معنی‌دار برای خود مراقبتی بودند و سازه‌های مدل اعتقاد بهداشتی در مجموع 33% از واریانس رفتار خود مراقبتی را توضیح می‌دادند. نتایج همچنین نشان داد که خود کارآمدی (37/0ß=) مهمترین پیشگویی کننده است. نتیجه گیری: نتایج مطالعه، رابطه بین منافع، موانع و خود کارآمدی درک‌شده با رفتارهای خود مراقبتی در بیماران دیابتی را تایید می‌کند، بنابراین ضرورت تقویت این سازه‌ها به خصوص در بیمارانی که در این حوزه ضعیف‌تر هستند پیشنهاد می‌شود.

Title: موانع درک شده برای انجام تست خون مخفی در مدفوع جهت غربالگری سرطان کولورکتال از دیدگاه افراد در معرض خطر متوسط شهر اصفهان

http://hsr.mui.ac.ir/index.php/jhsr/article/view/1251

مقدمه: پیشگیری ثانویه در سرطان کولورکتال امکان درمان و جلوگیری از پیشرفت بیماری را فراهم می کند. تست خون مخفی در مدفوع به دلیل سهولت انجام و همچنین هزینه اندک، بر دیگر روشهای غربالگری این بیماری اولویت دارد و انجام هرساله آن برای همه افراد بالای 50 سال توصیه شده است. پژوهش حاضر با هدف تعیین موانع مراجعه جهت انجام تست خون مخفی در مدفوع از دیدگاه افراد بالای 50 سال شهر اصفهان صورت پذیرفت. روش‌ها: این مطالعه از نوع مورد شاهدی است که بر روی 196 نفر از افراد بالای 50 سال شهر اصفهان انجام شد. دو گروه از افراد در این پژوهش مورد بررسی قرار گرفتند: نخست افراد مراجعه کننده به آزمایشگاه جهت انجام تست خون مخفی در مدفوع و گروه دوم افراد غیر مراجعه کننده به آزمایشگاه. اطلاعات مورد نیاز پژوهش از طریق پرسشنامه استاندارد شده جمع آوری شد. داده های حاصله از طریق نرم افزار SPSS و بکارگیری آزمون های آماری توصیفی و تی تست مورد تجزیه و تحلیل قرار گرفتند. یافته‌ها: نمره موانع درک شده در گروه نخست 09/40 و برای گروه غیر مراجعه‌کننده به آزمایشگاه 54/56 بود)001/0(P<. همچنین افرادی از کل جمعیت که سابقه انجام تست در سال گذشته را داشتند با میانگین نمره 04/35 در مقایسه با سایرین با میانگین 14/56، موانع کمتری را گزارش نمودند )001/0( p<. بیشترین موانع ذکر شده توسط گروهی از افراد که به آزمایشگاهها مراجعه ننموده بودند شامل: کمبود اطلاعات، ترس از تشخیص سرطان، عدم اولویت زمانی برای انجام این تست و عدم توصیه پزشک گزارش شد. نتیجه‌گیری: با توجه به یافته‌های این پژوهش اطلاع‌رسانی در سطح جامعه در مورد سرطان کولورکتال و همچنین ضرورت انجام تستهای غربالگری، بعلاوه ایجاد تسهیلات لازم جهت افزایش مشارکت افراد در انجام این مهم باید مورد توجه قرار گیرد.

Title: بررسی کاربرد رویکرد فرآیند عمل بهداشتی در ارتقای مهارت های فرزندپروری مادران

http://hsr.mui.ac.ir/index.php/jhsr/article/view/1274

مقدمه: برنامه فرزندپروری مثبت بارویکرد فرآیند عمل بهداشتی برای والدین کودکانی که در معرض ابتلا به اختلالات رفتاری و عاطفی قرار گرفته‌اند به کار می‌رود و هدف این پژوهش بررسی کاربرد رویکرد فرآیند عمل بهداشتی در ارتقای مهارت‌های فرزندپروری مادران بود. روش‌ها: این پژوهش بر روی مادران واجد فرزند 3 الی 8 سال انجام شد. حجم نمونه 120 نفر تخمین زده شد. روش نمونه‌گیری به صورت خوشه‌ای چند مرحله‌ای تصادفی بود. ابزار گردآوری داده‌ها، پرسشنامه طراحی شده بر اساس سازه‌های مدل رویکرد فرآیند عمل بهداشتی و آزمون آسکی بود. روایی محتوی و صوری پرسشنامه و چک لیست‌ها توسط پانل خبرگان تایید شد و روایی ساختاری هم با تحلیل عاملی تاییدی به دست آمد. پایایی ابزارهای اندازه‌گیری نیز توسط آلفا کرونباخ به دست آمد. اطلاعات جمع‌آوری شده با نرم‌افزار آماری SPSSIBM نسخه 21تحلیل گردید. یافته‌ها: آزمون کای دو نشان داد که بین دو گروه مداخله و غیر مداخله از نظر میانگین رفتار مهارت‌های فرزندپروری قبل و بعد از مداخله، اختلاف معنی‌دار آماری وجود دارد. با آزمون تی مستقل مشخص شد که بعد از مداخله آموزشی، میانگین نمره سازه‌های پرسشنامه در هر دو گروه اختلاف معنی‌داری داشت و نتایج آنالیز رگرسیون و رگرسیون لوجستیک نشان داد که سازه‌های مدل تبیین کننده 9/71-1/53 ٪ واریانس انجام مهارت‌های فرزندپروری بودند و مدل برازش خوبی را نشان داد. نتیجه‌گیری: با توجه به یافته‌های پژوهش چنین استنباط می‌شود که مداخله آموزشی‌ای که در چارچوب مدل رویکرد فرآیند عمل بهداشتی و بر اساس سازه‌های این مدل طراحی شده بود، تاثیر مثبتی در ارتقای مهارت‌های فرزندپروری مادران داشته و عملکرد آنها را به طور چشمگیری افزایش داده است. لذا شایسته است از این مدل در برنامه‌ریزی‌های آموزشی مربوط به آموزش خانواده خصوصا مهارت‌های فرزندپروری بهره گرفته شود.

Title: آموزش بالینی اثربخش از دیدگاه کارآموزان و کارورزان پزشکی

http://hsr.mui.ac.ir/index.php/jhsr/article/view/1225

مقدمه:آموزش بالینی اثربخش از بخش‌های اساسی در آموزش پزشکی محسوب می‌شود. بررسی دیدگاه دانشجویان این عرضه، می‌تواند گام مؤثری در تدوین برنامه آموزش بالینی باشد. این مطالعه با هدف تعیین دیدگاه دانشجویان کارآموز و کارورز پزشکی در خصوص آموزش بالینی اثربخش صورت گرفته است. روش‌ها:در این مطالعه مقطعی کلیه دانشجویان کارآموز و کارورز دانشکده پزشکی دانشگاه آزاد اسلامی واحد قم (140 نفر) شرکت کردند. داده‌ها توسط پرسشنامه محقق ساخته روا و پایا در 6 حیطه خصوصیات فردی فراگیر، مدرس بالینی، محیط بالینی، برنامه‌ریزی آموزشی، ارزشیابی بالینی و امکانات رفاهی بر اساس مقیاس نگرش سنج لیکرت گردآوری شد. تحلیل داده‌ها با نرم‌افزار SPSS به صورت توصیفی و تحلیلی بیان گردید. یافته‌ها:در این مطالعه، حیطه مؤثر بر آموزش بالینی به ترتیب، مدرس بالینی، خصوصیات فردی فراگیر، برنامه‌ریزی آموزشی، ارزشیابی بالینی، محیط بالینی و امکانات رفاهی بودند. همچنین مهمترین عامل مؤثر بر آموزش بالینی در حیطه خصوصیات فراگیر، اعتماد به نفس، در حیطه مدرس بالینی، تعهد و مسؤولیت‌پذیری، در حیطه محیط بالینی، نگرش مثبت به آموزش دانشجو، در حیطه برنامه‌ریزی آموزشی، کیفیت آموزش دانشجو، در حیطه ارزشیابی بالینی، وجود نظام ارزشیابی بالینی مدون و در حیطه امکانات رفاهی، وجود اتاق معاینه به دست آمد. بحث: دو حیطه مدرس بالینی و خصوصیات فردی فراگیر نسبت به سایر حیطه‌های دیگر از اهمیت بیشتری برخوردار بودند. لذا توجه به تقویت مدرسان بالینی در خصوص آموزش اثربخش و نیز تقویت علاقه و اعتماد به نفس دانشجویان در عرصه آموزش بالینی می تواند حائز اهمیت باشد.

Title: بررسی میزان آگاهی و نگرش پزشکان،پرستاران و بیماران در خصوص منشورحقوق بیماران در بیمارستان‌های وابسته به دانشگاه علوم پزشکی قم در سال1390

http://hsr.mui.ac.ir/index.php/jhsr/article/view/1258

مقدمه: یکایک افراد جامعه متعهد به حفظ و احترام به کرامت انسان‌ها می‌باشند. این امر در شرایط بیماری از اهمیت ویژه‌ای برخوردار است. آگاهی و رعایت حقوق بیمار از ارکان نظام سلامت کارآمد است. مطالعه حاضر به منظور تعیین آگاهی و نگرش پزشکان، پرستاران و بیماران در خصوص منشور حقوق بیماران در بیمارستان‌های وابسته به دانشگاه علوم پزشکی قم در سال1390 انجام گرفته است. روش‌ها: این مطالعه توصیفی – تحلیلی است که در پنج بیمارستان وابسته به دانشگاه علوم پزشکی قم انجام گرفت. بدین ترتیب که 283 نمونه ازپزشکان و پرستاران و بیماران به صورت در دسترس و تصادفی (بیماران) انتخاب شدند. ابزار گردآوری داده‌ها پرسشنامه محقق ساخته مشتمل بر 3 قسمت سؤالات دموگرافیک، سؤالات آگاهی (30 سؤال) و سؤالات نگرش (2 سؤال) بود. داده‌ها به وسيله آمار توصيفي- استنباطی با نرم‌افزار SPSS  نسخه 16 مورد تجزيه و تحليل قرار گرفت. یافته‌ها: میانگین نمره آگاهی (09/8 ± 22/25) و نگرش پرستاران (16/12 ± 21/63) به طور معنی‌داری از پزشکان و بیماران بیشتر (001/0>P) بود. حداقل نمره میانگین در خصوص سطح نگرش پزشکان و پرستاران به گویه« لزوم ارایه توضیحات به بیمار در مورد روند درمان روزانه» اختصاص یافت. نتیجه‌گیری: وجود فاصله بین وضعیت موجود و مطلوب سطح آگاهی و نگرش پزشکان و پرستاران می‌تواند در نهایت موجب عدم رعایت حقوق بیماران گردد. شناسايي موانع و راهكارهاي اجرايي رعايت حقوق بيماران توسط مسؤولین ضروری می‌نماید.

Title: بررسی نگرش و هنجارهای انتزاعی ترغیب کننده ماندگاری در درمان نگهدارنده با متادون

http://hsr.mui.ac.ir/index.php/jhsr/article/view/1634

مقدمه: ميزان ماندگاري در درمان نگهدارنده با متادون از شاخص هاي بسيار مهم در درمان اعتیاد می باشد. مطالعه حاضر با هدف تعیین نگرش و هنجارهای انتزاعی ماندگاری در درمان نگهدارنده با متادون در بین معتادان استان کهکیلویه و بویر احمد انجام گرفت. روش‌ها: این مطالعه یک پژوهش توصیفی مقطعی می باشد كه در بین معتادین مرد مراجعه کننده به مراکز ترک اعتیاد که در حال دریافت درمان نگهدارنده با متادون بودند، انجام شد. نمونه گیری به صورت سیستماتیک با انتساب متناسب از بین مراکز ترک اعتیاد موجود در هر یک از شهر های مختلف استان صورت گرفت. داده ها با بهره گیری از نرم افزار آماری SPSS ویرایش 21 مورد توصیف قرار گرفت. یافته‌ها: میانگین سن شرکت کنندگان 94/8±58/33 با دامنه 16 تا 53 سال بود. در بین سوالات سازه نگرش به ترتیب کاهش تاثیر آسیب مواد بر سلامتی، ارتباط بهتر با اطرافیان و کاهش هزینه های زندگی، و در بین سوالات سازه هنجارهای انتزاعی، تایید خانواده و تعداد دوستان درگیر متادون درمانی بیشترین میانگین را بدست آوردند. نتیجه‌گیری: به نظر می رسد افزایش باورهای نظیر کاهش آسیب، کاهش هزینه های زندگی و بهبود روابط و همچنین تاثیر حمایت های خانواده و دوستان برای ماندگاری بر درمان نگهدارنده با متادون در بین معتادان می تواند نقش موثری داشته باشد.

Title: تأثیر آموزش بر اساس مدل اعتقاد بهداشتی بر عملکرد زنان در مورد پاپ اسمیر

http://hsr.mui.ac.ir/index.php/jhsr/article/view/1285

مقدمه: سرطان دهانه رحم دومین سرطان شایع بین زنان است و این در حالی است که علی‌رغم اکثر سرطان‌ها این سرطان با انجام آزمایش پاپ اسمیر قابل تشخیص و پیشگیری است.اغلب سرطان‌های تهاجمی دهانه رحم در زنانی دیده می‌شود که به طور منظم اقدام به انجام پاپ اسمیر نکرده‌اند. این مطالعه اثرات برنامه آموزشی بر اساس مدل اعتقاد بهداشتی بر عملکرد زنان خمینی شهر در مورد اقدام به انجام پاپ اسمیر را بررسی کرده است. روش‌ها:این مطالعه به صورت مداخله‌ای آینده‌نگر بر روی 124 زن تحت پوشش دو مرکز بهداشتی درمانی خمینی شهر انجام شد که به طور تصادفی در دو گروه شاهد و مورد قرار گرفته و از نظر عملکرد و مشخصات دموگرافیک یکسان‌سازی شدند. ابزار گردآوری اطلاعات پرسشنامه بود که روایی و پایایی آن تأیید شده بود و شامل بخش‌های آگاهی،اجزای مدل و عملکرد و مشخصات دموگرافیک بود. پرسشنامه در دو مرحله قبل و یک ماه بعد از مداخله تکمیل شد. نتایج به وسیله نرم‌افزار SPSS نسخه 16 تجزیه و تحلیل و از آزمون‌های تی‌مستقل، تی‌زوج و کای اسکوئر استفاده شد. یافته‌ها: نتایج این مطالعه نشان داد که نمرات آگاهی زنان گروه مورد بعد از مداخله به طور معنی‌داری از گروه شاهد بیشتر بود (001/0P<). همچنین نمرات حساسیت درک شده، شدت درک شده، منافع درک شده و موانع درک شده گروه مورد بعد از مداخله به طور معنی‌داری نسبت به گروه شاهد بهبود داشت. (001/0P<) اقدام به انجام پاپ اسمیر نیز در گروه مورد(53 نفر یا 5/85٪) به طور معنی‌داری بعد از مداخله نسبت به گروه شاهد افزایش داشت (001/0P<). نتیجه‌گیری:یافته‌های این مطالعه نشان داد که آموزش بر اساس مدل اعتقاد بهداشتی تأثیر مثبت بر اقدام به موقع جهت انجام پاپ اسمیر داشته است.

Title: بررسی سطح آگاهی و عملکرد ایمنی کارکنان دانشگاه علوم پزشکی شیراز در ارتباط با مسمومیت با گاز منواکسید کربن ( گاز گرفتگی)

http://hsr.mui.ac.ir/index.php/jhsr/article/view/1582

چکیده : زمینه و هدف : منواکسید کربن گازی بی رنگ ، بی بو، بی مزه و غیر محرک است که در احتراق ناقص مواد کربن دار به ويژه در محيطهای بسته  تولید می شود اين مطالعه با هدف  بررسی  سطح آگاهی و عملکرد  ایمنی کارکنان افراد در مورد مسوميت با منوکسيد کربن انجام شد.  روش بررسی : این مطالعه بر روی 188 نفر از کارکنان دانشگاه علوم پزشکی شیراز انجام شد. ابزار مورد استفاده برای بررسی پرسشنامه ای ساختار یافته ای  متشکل از  سوالاتی در زمینه های  اطلاعات کلی، سطح آگاهی و عملکرد ایمنی بود که به روش خود گزارش دهی تکميل گرديد.  داده ها  با استفاده از نرم افزار آماری SPSS-13 و سطح معنی داری 05/0  مورد  تحلیل قرار گرفتند. یافته ها : اغلب افراد مورد بررسی  از سطح آگاهی بالایی  و سطح عملکرد ایمنی متوسطی برخوردار بودند. از بين متغیرهای مورد بررسی، ارتباط بین سن و عملکرد (008/0 p=) ، جنس و عملکرد (003/ 0 p=) و نیز  ارتباط بین تجربه ی عوارض خفیف گازگرفتگی و  سطح آگاهی ) 026/  0 P=) معنا دار بود.    نتیجه گیری : نتايج اين مطالعه نشان داد برای بهبود سطح عملکرد ایمنی افراد در مورد اقدامات مربوط به پیشگیری از مسموميت با منوکسيد کربن، لازم است برنامه های آموزشی و نظارتی بیشتری در اين زمينه انجام شود.

Title: بررسی تاثیر آموزش بر اساس مدل اعتقاد بهداشتی به منظور پیشگیری از مصرف خودسرانه دارو در زنان شهر سبزوار

http://hsr.mui.ac.ir/index.php/jhsr/article/view/1440

مقدمه: خوددرمانی و مصرف خودسرانه دارو امروزه یکی از معضلات بهداشتی محسوب می‌گردد که در جامعه زنان به دلیل داشتن دوران‌های حساسی همچون بارداری و شیردهی و مسؤولیت خانه و خانواده که آگاهی و نگرش آن‌ها برای دیگر اعضای خانواده مهم تلقی می‌گردد ازاهمیت مضاعفی برخوردار است. لذا مطالعه حاضر با هدف تاثیر آموزش بر اساس مدل اعتقاد بهداشتی به منظور پیشگیری از مصرف خودسرانه داروها در زنان مراجعه کننده به مراکز بهداشتی درمانی شهر سبزوار صورت گرفته است. روش‌ها: مطالعه حاضر مداخله‌ای و از نوع نیمه تجربی (شاهددار) قبل و بعد می‌باشد و جهت مطالعه آینده‌نگر است. در این مطالعه 160 نفر از زنان مراجعه کننده به مراکز بهداشتی درمانی شهر سبزوار به صورت تصادفی ساده انتخاب و در دو گروه مورد و شاهد هر کدام 80 نفر وارد مطالعه شدند. روش گردآوری اطلاعات پرسش‌نامه تنظیم شده شامل سؤالات آگاهی، نگرش (براساس اجزای مدل اعتقاد بهداشتی)، چک لیست عملکرد و علل خوددرمانی در خصوص مصرف خودسرانه دارو بوده است. قبل از انجام مداخله آموزشی پرسش‌نامه و چک لیست و علل خوددرمانی برای هر دو گروه مورد و شاهد تکمیل و سپس مداخله آموزشی برای گروه مورد در طی 3 هفته و در قالب 3 جلسه آموزشی 60 دقیقه‌ای انجام گرفت. جهت تجزیه و تحلیل داده‏ها از نرم افزار آماریSPSS نسخه 16، آزمون‏های آماری تی‌تست زوجی، آزمون مجذور کای و ویلکاکسون استفاده شد، نتایج در سطح 05/0> Pمعنی‌دار تلقی گشت. یافته‌ها: یافته‌های پژوهش نشان داد که در قبل از مداخله آموزشی آگاهی و نگرش زنان در زمینه خوددرمانی در حد متوسط و عملکرد آنان در زمینه مصرف خودسرانه داروها ضعیف بود و این مقدار در گروه مورد و شاهد بدون ارتباط معنی‌دار بود (72/0 = P). ولی پس از مداخله آموزشی بین دو گروه مورد و شاهد در همه متغیرهای ذکر شده اختلاف معنی‌داری ایجاد شد (041/0=P) و عملکرد زنان در زمینه مصرف خودسرانه دارو کاهش پیدا کرد. نتیجه‌گیری: آموزش برمبنای مدل اعتقاد بهداشتی در ارتقا آگاهی و نگرش و عملکرد زنان مراجعه کننده به مراکز بهداشتی درمانی در رابطه با پیشگیری از مصرف خودسرانه داروها اثربخش بود. در نتیجه انجام مداخلات آموزشی با به کارگیری مدل‌های آموزش بهداشت به خصوص مدل اعتقاد بهداشتی در پیشگیری وکاهش مصرف خودسرانه دارو و بهبود رفتار بهداشتی خوددرمانی توصیه می‌گردد.

Title: بررسی میزان تأثیر آموزش بر دانش و نگرش مدیران مدارس ابتدایی شهرستان برخوار در زمینه استقرار سامانه مدیریت ایمنی بهداشت و محیط زیست ( Health safety and Environmental Management System)

http://hsr.mui.ac.ir/index.php/jhsr/article/view/1278

مقدمه: وجود معضلات بهداشتی و جنبه‌های خطر ساز و نیز پیامدهای زیست محیطی که روز به روز ذهن کارشناسان و دست‌اندرکاران سلامت و محیط زیست را به خود مشغول کرده باعث گردید تا دولت‌ها در پی راه چاره بوده و امکانات خود را در راه بهبود وضعیت بهداشتی و زیست محیطی جوامع به کار گیرند. وجود آگاهی و دانش مدیریت سلامت در مسؤولین مهمترین رکن در ارتقا و حفظ بهداشت محیط مدارس و ایمنی دانش‌آموزان است. هدف این مطالعه تأثیر آموزش بر دانش و نگرش مدیران مدارس ابتدایی در زمینه استقرار سامانه مدیریت ایمنی بهداشت و محیط زیست است. روش‌ها: در این مطالعه که از نوع کاربردی و به روش شبه آزمایشی انجام گردید مدیران مدارس ابتدایی به تعداد 46 نفر به  عنوان جمعیت مورد مطالعه انتخاب شده و کارگاه آموزشی 2 روزه برای آنها برگزار شد. قبل از اجرای کارگاه آموزشی توسط پرسش‌نامهمحقق ساخته دانش و نگرش آنان سنجیده شد و پس از اجرای کارگاه آموزشی نیز دانش و نگرش آنها توسط همان پرسش‌نامهها محاسبه گردید و داده های به دست آمده توسط نرم افزار SPSS نسخه 20 و آزمون t تجزیه و تحلیل گردید. یافته‌ها: میانگین نمره حاصل در پرسش‌نامهدانش در سه زمینه بهداشتی ایمنی، سیستمی و محیط زیستی قبل از مداخله آموزشی 45 به دست آمد که پس از انجام مداخله به 82 ارتقا پیدا کرد به عبارت دیگر از سطح ضعیف به سطح خیلی خوب رسید و در زمینه نگرش مدیران نیز میانگین نمره آنها قبل از مداخله در سه زمینه فوق الذکر73 بود که پس از مداخله آموزشی به 81 ارتقا پیدا کرد. به عبارت دیگر از سطح خوب به خیلی خوب رسید. نتیجه‌گیری: هدف از استقرار سامانه مدیریت ایمنی بهداشت و محیط زیست در یک سازمان حفظ سلامتی کارکنان آن سازمان می باشد.  مدرسه نیز به عنوان مصداق یک سازمان نیازمند چنین سامانه ای است . به ویژه این نکته که در بردارنده وظیفه خطیر آموزش و پرورش دانش آموزان می باشد. در این پژوهش به وضوح نقش آموزش در ارتقای سطح دانش، نگرش مدیران مدارس ابتدایی در زمینه استقرار سامانه مدیریت ایمنی بهداشت و محیط زیست  نشان داده شده است.

Title: تأثير آموزش بهداشت بر مبناي نظريه رفتار برنامه ريزي شده در ارتقاء رفتارهاي ايمن عبور از خيابان در دانش آموزان

http://hsr.mui.ac.ir/index.php/jhsr/article/view/1257

مقدمه: آسيب‌هاي عابرين پياده و کودکان يکي از دلايل مهم مرگ و ناتواني است و پيشگيري از اين امر نيازمند راهبردهاي آموزشي و اجراي مداخلات جامع مي‌باشد. این مطالعه با هدف بررسي تأثير مداخله آموزشی بر مبناي نظريه رفتار برنامه‌ريزي شده در ارتقا رفتارهاي ايمن عبور از خيابان دانش‌آموزان چهارم ابتدايي شهر تهران انجام گرديد. روش‌ها: مطالعه به صورت کارآزمايي شاهددار تصادفي بر روي 160 دانش‌آموز چهارم ابتدايي(80 دختر و80 پسر) که به صورت تصادفي از مدارس منطقه 4 شهر تهران انتخاب شده بودند، در دو گروه آزمون و کنترل انجام شد. بعد از تعيين نيازها در چارچوب نظريه رفتار برنامه‌ريزي شده، مداخله آموزشي طراحي و براي گروه آزمون به اجرا در آمد. 2 ماه پس از اتمام مداخله آموزشی هر دو گروه پيگيري و در نهايت داده‌ها با استفاده از نرم‌افزار آماريي SPSS نسخه 16 مورد تجزيه و تحليل قرارگرفت.   يافته‌ها: در مرحله قبل از مداخله بين دو گروه از نظر متغيرهاي دموگرافيک، رفتار و سازه‌هاي نظريه رفتار برنامه‌ريزي شده که شامل، قصد رفتاري، توانمندی درک شده، اعتقادات کنترلی، انگیزه پیروی، اعتقادات هنجاری، ارزشیابی پیامد و اعتقادات رفتاری بودند، تفاوت معني‌دار وجود نداشت. پس از انجام مداخله، بين دو گروه آزمون و کنترل از نظر سازه‌هاي قصد رفتاري          (0001/0P<) ، توانمندي درک شده (0001/0 P<)، اعتقادات کنترلي (0001/0P<) ، انگيزه پيروي (0001/0P<)، اعتقادات هنجاري (0001/0P<)، ارزشيابي پيامد (0001/0P<)، اعتقادات رفتاري (0001/0P<) و رفتارهاي ايمن عبور از خيابان          (0001/0P<) تفاوت معني‌داري مشاهده شد. نتيجه‌گيري: يافته‌ها نشان داد که مداخله آموزشي بر مبناي نظريه رفتار برنامه‌ريزي شده مي‌تواند سبب ارتقا رفتارهاي ايمن عبور از خيابان دانش‌آموزان گردد.

Title: نيازسنجي آموزشي کارکنان تحت پوشش دانشكده علوم پزشكي دزفول در زمینه دوره هاي آموزش ضمن خدمت

http://hsr.mui.ac.ir/index.php/jhsr/article/view/1189

مقدمه: دوره‌های آموزشی ضمن خدمت، کارکنان سازمان را در حل مشکلات، اخذ تصمیمات و تجزیه و تحلیل شغلی تواناتر ساخته است. برنامه‌هاي آموزشي كاركنان باید بر اساس نيازسنجي آموزشي انجام شود. لذا مطالعه حاضر با هدف تعيين نيازسنجي آموزشي کارکنان دانشكده علوم پزشكي دزفول در دوره‌هاي آموزش ضمن خدمت در سال 1391 انجام شد. روش‌ها: مطالعه حاضر به صورت توصيفي، تحليلي بر روي 1800 نفر از کارکنان دانشكده علوم پزشكي دزفول انجام شد. نمونه‌گيري به صورت غير تصادفي و مبتني بر هدف بود. ابزار گرد‌آوری داده‌ها پرسشنامه محقق ساخته مشتمل بر اطلاعات جمعیت‌شناختی و سؤالات تخصصی در حيطه‌های مهارتی و مشکلات اجرایی بود. جهت تعیین اعتبار و پايايي پرسشنامه به ترتيب از اعتبار محتوي و آزمون مجدد استفاده شد. اطلاعات به وسیله نرم‌افزار SPSS ویرایش 15 مورد تجزیه و تحلیل قرار گرفت. یافته‌ها: یافته‌ها نشان داد که بالاترين نياز آموزشي در بین کلیه حیطه‌های آموزشی، مربوط به مهارت‌هاي فنی و عملیاتی (01/1±18/4) و کمترين آن مربوط به مهارت هاي نظری و ادراکی (89/0±87/3) بود. بيشترين نياز آموزشي در حیطه مهارت‌هاي فني، به رسته بهداشتی درمانی، در مهارت‌هاي نظری، به رسته اداری مالی و در مهارت‌هاي ارتباطی ، به رسته بهداشتی درمانی اختصاص داشت. نتيجه‌گيري: مهارت‌هاي فنی و عملیاتی و نیازهای روزانه کارکنان، مهم‌ترين نيازهاي کارکنان محسوب می‌شود و لازم است در تنظيم برنامه‌هاي ساليانه مد نظر قرار داده شود.

Title: تأثیر آموزش مهارت‌های اجتماعی بر خودکارآمدی پرهیز از مواد مخدر در نوجوانان پسر دبیرستان‌های اهواز

http://hsr.mui.ac.ir/index.php/jhsr/article/view/1466

مقدمه: کاهش سن اعتیاد و رشد سریع وابستگی به مواد در افراد زیر 18سال، اولویت آموزش مهارتهای اجتماعی جهت پرهیز از مواد در این گروه را آشکار می سازد.  مطالعه حاضر به سنجش تاثیر آموزش مهارتهاي اجتماعي برخودكارآمدي پرهيز از مواد مخدر در دانش آموزان پسر شهر اهواز پرداخته است. روش‌ها: این پژوهش نیمه تجربی برروی دانش آموزان پسر پایه اول دو دبیرستان ناحیه 3 شهر اهواز با تخصیص تصادفی در سال تحصیلی 92-91 انجام شد. در هریک از مدارس آزمون و کنترل 60 نفر(جمعاً120نفر) به روش تصادفی انتخاب شدند. مداخله فقط درگروه آزمون، شامل آموزش مهارتهای اجتماعی در پرهیز از مواد مخدر باتلفیق سازه خودکارآمدی بندورا و مبتنی بر یادگیری فعال بود. هر دو گروه قبل و بعد از مداخله به پرسشنامه های خودکارآمدی پرهیز از مواد مخدر و مهارتهای اجتماعی پاسخ دادند. داده ها با آزمون تی مستقل و زوجی آنالیز گردید. یافته‌ها: میانگین خودکارآمدی آن پرهیز از مواد مخدر بین پسران دو گروه آزمون وکنترل در پیش آزمون تفاوت معناداری نداشت، اما در پس آزمون بین میانگین های دو گروه  تفاوت معناداری مشاهده گردید(05/0P<). مداخله آموزش موجب افزایش قابل توجه و معنادار میانگین نمره مولفه خودکارآمدی در موقعیت اجتماعی از97/2±36/18 به 43/1±73/19 (001/0=P) و خودکارآمدی در موقعیت هیجانی از30/10±83/58 به 98/2±85/63 (001/0=P) شد. نتیجه‌گیری: مداخله آموزشی در افزایش خودکارآمدی پرهیز از مواد مخدر به ویژه در موقعیت اجتماعی و هیجانی موثر بوده است. لذا آموزش مهارت‌های اجتماعی با هدف ارتقا خودکارآمدی پرهیز از مواد مخدر در موقعیت های فوق، می تواند به منزله رهیافت مقابله ای در آموزش نوجوانان مدنظر قرار گیرد.

Title: پیش بینی عوامل موثر بر عود مصرف مواد مخدر در بین بیماران تحت درمان نگهدارنده با متادون درمراکز درمان اعتیاد شهر همدان با بهره گیری از مدل اعتقاد بهداشتی

http://hsr.mui.ac.ir/index.php/jhsr/article/view/1534

مقدمه: یکی از چالش‌های عمده کنونی در درمان نگهدارنده با متادون میزان ماندگاری بر درمان یا پیشگیری از عود اعتیاد می‌باشد. مطالعه حاضر با هدف تعیین عوامل موثر بر عود اعتیاد در بیماران تحت درمان نگهدارنده با متادون مبتنی بر مدل اعتقاد بهداشتي در مراکز درمان اعتیاد شهر همدان صورت گرفت. روش‌ها: این مطالعه توصيفي مقطعی در بهار و تابستان سال 1392 بر روي 384 نفر از بيماران تحت درمان نگهدارنده با متادون در مراکز درمان اعتیاد شهر همدان انجام شد. نمونه‌گیری به صورت طبقه‌بندی با انتساب متناسب انجام گرفت. ابزار جمع‌آوري داده‌ها پرسشنامه طراحي شده بر اساس سازه‌هاي مدل اعتقاد بهداشتي و متغيرهاي دموگرافيك بود و پرسشنامه‌ها به صورت خودگزارش‌دهی و مصاحبه تکمیل شد. تجزیه و تحلیل داده‌ها با استفاده از نرم‌افزار SPSS نسخه 19 و بهره‌گیری از آنالیز رگرسیون و کای دو صورت گرفت. یافته‌ها: میزان عود در بیماران تحت درمان نگهدارنده با متادون 2/48٪ بود. وسوسه، فشار دوستان، افسردگي و کم بودن دوز دارویی متادون از مهمترین دلایل عود بودند. آنالیز رگرسیون نشان داد سازه‌هاي حساسیت درك شده، منافع درک شده و خودكارآمدي پیش‌بینی‌کننده‌های قوی‌تری برای عود بودند. نتیجه‌گیری: با توجه به نتایج، به نظر می‌رسد مدل اعتقاد بهداشتی بتواند در برنامه‌های مداخله‌ای پیشگیری از عود کاربرد داشته باشد.

Title: بررسی اثربخشی آموزش روان‌درمانی مبتنی بر نظریه فِریش بر کيفيت زندگي مراجعان شهر اصفهان

http://hsr.mui.ac.ir/index.php/jhsr/article/view/1279

مقدمه: کيفيت زندگي افراد نقش تعيين‌کننده‌اي در رضايت آنان از زندگي ايفا مي‌کند و رضايتمندي از زندگي عامل مؤثري در بهداشت رواني افراد جامعه مي‌باشد. بنابراين شناخت روش‌ها و شيوه‌هاي آموزش کارآمد و مؤثري که بتواند در جهت افزايش کيفيت زندگي افراد مؤثر واقع شود گامي مهم و اساسي در ارتقا رضايتمندي و در نتيجه افزايش بهداشت رواني آنان محسوب مي‌گردد. لذا هدف از انجام اين پژوهش بررسي اثربخشی آموزش روان‌درمانی مبتنی بر نظریه فِرِش بر کيفيت زندگي افراد مراجعه‌کننده به مراکز مشاوره شهر اصفهان بود. روش‌ها: پژوهش حاضر نیمه تجربي از نوع پيش‌آزمون، پس‌آزمون با گروه کنترل بوده، جامعه آماري اين پژوهش افراد مراجعه کننده به 3 مراکز مشاوره شهر اصفهان (مرکز مشاوره دولتی دانشگاه صنعتی، مرکز مشاوره بوستان، مرکز مشاوره الفبای زندگی) در سال 1391 بودند. نمونه پژوهش شامل 32 افراد مراجعه کننده بود که از بين افراد در دسترس انتخاب شده و در دو گروه آزمايش و کنترل قرار داده شدند. مراجعه کنندگان گروه آزمايش، به مدت 8 جلسه در هشت هفته تحت آموزش به شيوه کيفيت زندگي درماني قرار گرفتند و در پايان از هر دو گروه پس آزمون به عمل آمد. ابزار اندازه‌گيري، پرسشنامه كيفيت زندگي سازمان بهداشت جهاني (WHOQOL- BREF) بود. داده‌ها توسط نرم‌افزار SPSS نسخه 18 و روش آماري تحليل کواريانس مورد تجزيه و تحليل قرار گرفت و 05/0<P از نظر آماری معنی‌دار تلقی شد. يافته‌ها: نتايج به دست آمده نشان داد که تفاوت معنی‌داری بين نمرات کيفيت زندگي گروه آزمايش و گروه گواه در مرحله پس‌آزمون وجود دارد. به اين معني که آموزش کيفيت زندگي درماني بر کيفيت زندگي افراد مؤثر بوده است (01/0P<). همچنين نتايج نشان داد آموزش کيفيت زندگي درماني، نمرات افراد را در پس‌آزمون گروه آزمايش در ابعاد سلامت جسمانی، سلامت رواني، روابط اجتماعي و سلامت محيط افزايش داده است (05/0P<). نتيجه‌گيري: نتايج اين پژوهش تأييدکننده تأثير مثبت آموزش رویکرد مبتنی بر نظریه فریش بر کيفيت زندگي افراد و افزايش‌دهنده رضايتمندي آنان از زندگي بود.

Title: بررسی علل مرگ و میر مادران در فاصله‏ ی سال ‏های 1390-1385 در استان اصفهان

http://hsr.mui.ac.ir/index.php/jhsr/article/view/2170

مقدمه :کاهش میزان مرگ و میر مادران بدون آگاهی از علل و میزان دقیق آن امکان‌پذیر نمی‌باشد. در این مطالعه با تعیین علل مستقیم و غیرمستقیم مرگ و میر مادران ، بررسی گزارش‌های اتوپسی و مراقبت‌های بهداشتی به بررسی دقیق این موضوع در استان اصفهان در سال‏های1390-1385 می‌پردازیم. مواد و روش ها: این مطالعه توصیفی تحلیلی و از نوع مقطعی است. .جمعیت مورد مطالعه مادران بارداری بودند که از شروع سال 85 تا پایان سال 90 طی دوران بارداری تا 42 روز پس از زایمان در استان اصفهان فوت کردند. لیست بیماران از معاونت درمان اخذ شد و پرونده هر بیمار بررسی شد. گزارش اتوپسی و صورت جلسه کمیته‌ مرگ و میر مادران باردار مطالعه شد. یافته‌ها :در 67 مورد مطالعه شده بیشترین آمار مربوط به سال 89 با 14 مورد می‌باشد. بیشترین علت فوت بر اساس یافته‌های بالینی و گزارش اتوپسی، خونریزی ( آتونی ) می‌باشد و بین این دو علت هماهنگی معنادار بود      ) 001/0P<).72% علل مرگ مستقیم، شامل خونریزی پس از زایمان (6/32٪)، عوارض ناشی از فشار خون (26٪)، ترومبوآمبولی (3/17٪) و 28% علل مرگ، غیرمستقیم که بیماری قلبی با3/47 % شایع‌ترین عامل غیرمستقیم مرگ بوده است. بیشترین مشکل در مراقبت‌های کل دوران بارداری، نامطلوب بودن خدمات ارائه شده، اشکال در فوریت‌های زایمانی و ثبت ناکامل اطلاعات بوده است. نتیجه‌گیری: در این مطالعه با توجه به  تمام عوامل درگیر، بیشترین میزان مرگ مربوط به خونریزی، عوارض ناشی از فشار خون و بیماری قلبی بوده است. کاهش این میزان می‌تواند از طریق آموزش و افزایش سطح آگاهی جامعه، بهبود پوشش و خدمات ارایه شده طی مراقبت‌های کل دوران بارداری، ارتقا تجهیزات و بهبود خدمات در دوران زایمان تحقق یابد.
